# Effective of prompt and delayed gamma emission on the nuclear radiation shielding effectiveness of prevalent natural minerals

**DOI:** 10.1038/s41598-025-24829-4

**Published:** 2025-11-10

**Authors:** Islam M. Nabil, Said M. Kassem, A. F. El-Sayed, Soliman M. El-Talawy, Atef El-Taher

**Affiliations:** 1 Physics Department, Faculty of Science, Fayoum University, Fayoum, Egypt; 2 Radiation Protection and Dosimetry Department, National Center for Radiation Research and Technology (NCRRT), Egyptian Atomic Energy Authority (EAEA), Cairo, Egypt; 3 Physics Department, Faculty of Science, Cairo University, Cairo, Egypt; 4 Physics Department, Faculty of Science, Azhar University, Cairo, Egypt; 5 Physics Department, Faculty of Science, Al-Azhar University, Assuit, 71524 Egypt

**Keywords:** Natural minerals, MCNP, EpiXs, Radiation shielding, Environmental sciences, Materials science, Physics

## Abstract

This study aims to explore the neutron and emphasizing gamma emission (prompt and delayed) radiation attenuation capabilities of the different natural minerals, including Magnetite, Rutile, Ilmenite, and concrete NBS materials. The Monte Carlo N–Particle simulation code (MCNP) and the EpiXs software (EpX) have been used to inspect the studied compounds against neutrons associated with secondary energetic γ-rays at the range 0.03–15 MeV of energy through different thicknesses 2–12 cm. For all materials, linear attenuation coefficients (µ), effective atomic number (Z_ef_), half-value layer (H_0.5_), tenth value layer (T_0.1_), mean free path (MFP), and radiation protection efficieny (RP_E_) are estimated. Additionally, fast neutrons flux, secondary γ-rays, the cross-section for the removal of fast neutrons (Σ_R_), the half-value thickness (HVLn), and the relaxation length (λn) were accurately calculated. The results show that Magnetite minerals are better than Rutile, Ilmenite, and concrete NBS minerals at lower energies zones. Moreover, it is more effective in providing protection against intense photons, fast neutrons, and secondary produced γ-rays, which makes this mixture an potentional common shield for a combination of various environmental radiation.

## Introduction

Nuclear power plants, nuclear reactors, industries, farms, radiopharmaceuticals, radiology, nuclear medicine, and many more fields rely on nuclear science. These uses emit various kinds of radiation^1,2^. When gamma, X-ray, or neutron radiation is present, every living thing, including humans, animals, and the environment, is put in danger. These factors have aroused the interest of a great number of scientists who are investigating possible substitutes for the radiation attenuation and shielding materials that are now in use^3^. It is mutual knowledge that shielding materials serve the purpose of reducing the radiation hazardous that occupational workers are exposed to by acting as a barrier among radioactive sources and employees or the surrounding region. The field of material science is concerned with gaining an understanding of the characteristics and behaviours of a wide range of materials at a variety of scales, including atomic scales, nanoparticles, and other levels^4,5^. This information is utilized in the process of designing and developing innovative, intelligent, and imaginative materials that possess particular features and characteristics that can be utilized in a variety of technological domains.

During the process of radiation shielding, in which the characteristics of materials are utilized to protect against the harmful effects of ionizing radiation, an application like this can be utilized to provide protection against the harmful effects of radiation. For the aim of radiation shielding, a wide variety of materials are now being utilized. These materials include concrete, cement, gypsum, borate glass, polymers, lead compounds, and a great deal of other materials. These materials are utilized in ways that are either conventional or sophisticated^6–10^. Therefore, in order to produce radiation shielding concrete (RSC) with the appropriate mechanical and physical qualities, scientists have experimented with a number of different methods to modify the mix design of concrete. This is due to the fact that concrete is the material that is used in construction the most frequently. Due to the fact that RSC is frequently used in nuclear facilities and is required to with stand both static and dynamic loads, this is of the utmost importance^11–14^.

Some of the alternatives to conventional coarse aggregates that have been the subject of extensive research include hematite, magnetite, galena, ilmenite, rutile, serpentine, silica, tourmaline, and steel slag. It is important to note that these alternatives have been explored^15–17^. Several academics have also considered incorporating certain synthetic or natural finely powdered components into the fine aggregates or using them as additives^18–21^.

In this situation, it becomes crucial to build concrete aggregate by substituting a different kind of aggregate for regular aggregate. One of the better options for this could be the study of neutrons and the characteristics of both prompt and delayed gamma radiation shielding properties, Prompt gamma affects real-time shielding performance while, Delayed gamma impacts long-term safety (e.g., nuclear waste storage, decommissioning). Studying neutron interactions and gamma emission (prompt/delayed) ensures optimal shielding performance, especially for natural minerals used in sustainable and cost-effective radiation protection. Recent research confirms their viability in nuclear reactors, medical radiation therapy, space exploration, and radioactive waste management applications.

The current research investigates the neutron and gamma emission (prompt/delayed) attenuation capabilities of natural minerals, including magnetite, rutile, ilmenite, and concrete NBS. In order to accomplish this objective, the linear attenuation coefficient (µ) has been evaluated by employing the Monte Carlo N–Particle simulation code (MCNP) and the EpiXs software (EpX). This evaluation was carried out based on a number of attenuation parameters, such as the effective atomic number (Z_ef_) and the radiation protection efficiency (RP_E_) at range 0.03–15 MeV of energy (γE). In addition to that, the cross-section for the removal of fast neutrons (Σ_R_), the half-value thickness (HVLn), and the relaxation length (λn) were accurately calculated. The primary objective of this stage is to investigate the effectiveness of the compounds that have been studied, particularly in an environment with mixed radiation (netruons and gamma-rays).

## Methodology

### Shielding materials

In the current study, natural minerals and concrete NBS were chosen to investigate the radiation attenuation capabilities shown by these materials. The three natural minerals, magnetite^22^, rutile^23^, ilmenite^24^, and concrete NBS^25^, were assessed. Table 1 display the densities and elemental fractional abundances of the investigated materials.‎

**Table 1:** Elemental composites and the densities of the investigated minerals.‎.

Composition, wt%				
Elements	Magnetite	Rutile	Ilmenite	Concrete NBS
O	0.4566	0.3885	0.3631	0.4983
Mg	0.0070	–	0.0043	0.0024
Al	0.0112	–	0.0054	0.0456
Si	0.0154	–	0.0035	0.3158
Ca	0.0036	–	0.0037	0.0826
Ti	0.0377	0.5417	0.2669	–
V	0.0029	0.0131	0.0012	–
Cr	0.0026	–	0.0019	–
Mn	0.0049	–	0.0092	–
Fe	0.4583	–	0.3396	–
Zr	–	0.0521	–	–
Nb	–	0.0047	–	–
P	–	–	0.0012	–
H	–	–	–	0.0056
Na	–	–	–	0.0171
K	–	–	–	0.0192
S	–	–	–	0.0012
Density (g/cm^3^)	5.15	4.25	4.72	2.25

### γ-ray attenuation theory

Identifying shielding materials can be accomplished by the utilisation of Lambert–Beer’s equation to compute the linear attenuation that is present^26,27^.

1 $$I=I_{o} e^{-\mu x}$$

where “I” represents the intensity of γ-rays that have passed through the material. “x” denotes the attenuator’s thickness, “µ” is the linear attenuation coefficient, and “I_o_” denotes the intensity of the primary gamma-rays in air.

It is possible to determine the mass attenuation coefficient (µ_m_), which is a relevant measurement of a material’s capability to attenuate particles, by utilising the formula that is provided at the end of this sentence^28,29^:

2 $$\mu_{m}=\frac{{\upmu\:}}{{\uprho\:}}$$

Understanding the µ parameter is crucial for figuring out what changes must be applied to the primary radiation to cut it in half or a tenth of its original value. By utilizing the following formulae, it is possible to achieve the half value layer (H_0.5_), which reduces the strength of the photon by half, and the absorber thickness that refers to the tenth value layer (T_0.1_), which reduces it to one-tenth^30^:

3 $$H_{0.5}\:=\:\frac{\text{l}\text{n}2}{{\upmu\:}}\:\:\:\:$$

4 $$T_{0.1}=\:3.32\: H_{0.5}$$

Equation 5 states that the mean free path (MFP) is the average path a photon travels prior to coming into contact with the absorber material^31^:

5 $$\:\text{M}\text{F}\text{P}=\:\frac{1}{{\upmu\:}}\:\:\:\:$$

Additionally, when assessing the barriar amount that may be offered by a variety of shielding materials, it is essential to take into consideration the radiation protection efficiency (RP_E_), which is a significant statistic. In addition, the transfer factor (TF) is described by Eq. 6, which states that it is the average intensity of the initial photons γ-rays that have travelled through the substance^32^.

6 $$\:\text{T}\text{F}=\:\frac{\text{I}}{\text{I}\text{o}}\:\:\:$$

7 $$\:\text{R}\text{P}\text{E}=\:1-\text{T}\text{F}\:\:\:$$

### Neutrons attenuation theory

When it comes to evaluating the neutron block possible of suggested shield materials, the fast neutrons removal cross-section (Σ_R_) is an essential instrument that plays a critical role. When taking into consideration the fact that the W_i_ represents the partial density and the ith signifies the mass cross-section of the constituent^33,34^:

8 $$\:{{\Sigma\:}}_{\text{R}}=\sum\:_{\text{i}}{\text{W}}_{\text{i}}$$

Furthermore, with regard to ascertain the half value layer (HVLn) and relaxation length (λn), the following formulas were utilised. In accordance with the relaxation length, the relaxation length is defined as the average distance that a fast neutron is able to travel into shield medium before it interacting particals of this medium^35,36^:

9 $$\:{\text{H}\text{V}\text{L}}_{\text{n}}\:=\:\frac{\text{l}\text{n}2}{{{\Sigma\:}}_{\text{R}}}$$

10 $$\:{{\uplambda\:}}_{\text{n}}=\frac{1}{{{\Sigma\:}}_{\text{R}}}$$

### MCNP simulation code

**Fig. 1:**
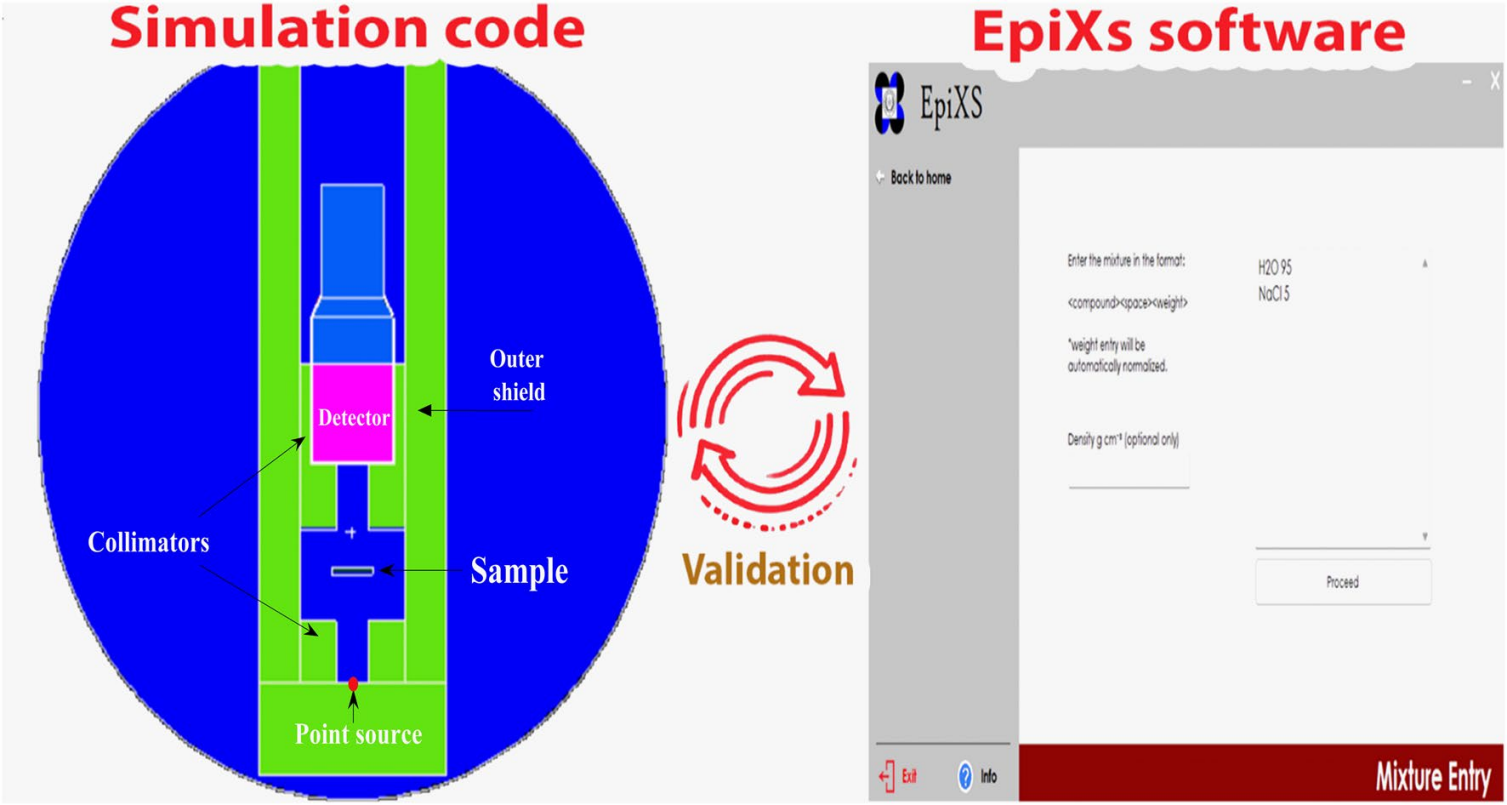
The geometry profile of the MCNP simulation code and EpiXs software.

Irradiation models of the studied mineral compounds were created and run in 0.030 ≤ γE ≤ 15 MeV using the MCNP code version 5, considering the principles of radiation interaction with matter^37^. The MCNP input files requirement particular information (e.g., source data, source location, sample/source geometry, densities, chemical compositions, etc.). The MCNP text input files consist of cell, surface, material, mode, and tally cards^38,39^. Regarding the surface card, it is used to describe the boundaries and dimensions of each cell. The cell is considered the smallest unit in geometry, where each consists of many cells arranged together. Besides, the material card is used to describe the chemical composition and density of each cell used in the geometry. The elemental composition and the density of minerals samples are listed in Table 1. The cell card described the system components: the radioactive source, γ-ray’s collimators, the attenuator sample, the outer shield, and the tally region. A monoenergetic γ-rays point source (source card) was created as ray/input file in the range 0.03–15 MeV. The same system configuration for the neutron attenuation is used except for the definition source of the neutron. It was designated as a fission source at 1 ≤ _n_E ≤ 12 MeV for the fast neutrons^40,41^. A cubic layer was constructed out of the samples, with the center of the layer being located between the origin and the counting zone. It was a lead collimator that was set up in the tally region that was responsible for focusing secondary γ-rays. The dimensions of the simulated system that was created with a γ/neutron ratio are displayed in Fig. 1. In addition, the secondary γ-rays that were emitted by the interactions between the neutrons were identified. With the help of the simulation code, a complete spectrum of the γ/neutron was obtained. The detector is an F4 tally to evaluate the mean track length over the sample cell. The calculation of the average track length of primary neutrons and gamma-rays is accomplished by utilizing the counts F4P and F4N (“In MCNP, F4P and F4N are the photon and neutron cell-flux (track-length) tallies; multiplying each tally by the cell volume yields the average track length in that cell per source particle’’). MCNP simulations were run with number of particles (NPS) = 1 × 10^7^ (ten million) particle histories per run; under this sampling, the tally relative errors were below 1%.

### EpiXs software

The Windows application known as EpiXs (EpX) is responsible for calculating a large number of factors that are associated with the attenuation and shielding of the compositions of the minerals that are being analysed (Fig. 1). A multitude of calculations were carried out, which included calculations for the linear attenuation coefficients (µ), the effective atomic number (Zef), and other related computations. As EpX input parameters, the oxide composition and densities of the MeV were included^42,43^. Furthermore, by contrasting the outcomes from EpX with the values from MCNP, the relative differences (φ, %) were determined^44^:

11 $$\varphi,(\%)\left\vert\frac{\text{M}\text{C}\text{N}\text{P}-\text{E}\text{p}\text{X}}{\text{M}\text{C}\text{N}\text{P}}\right\vert \times 100.$$

## Results and discussion

**Fig. 2:**
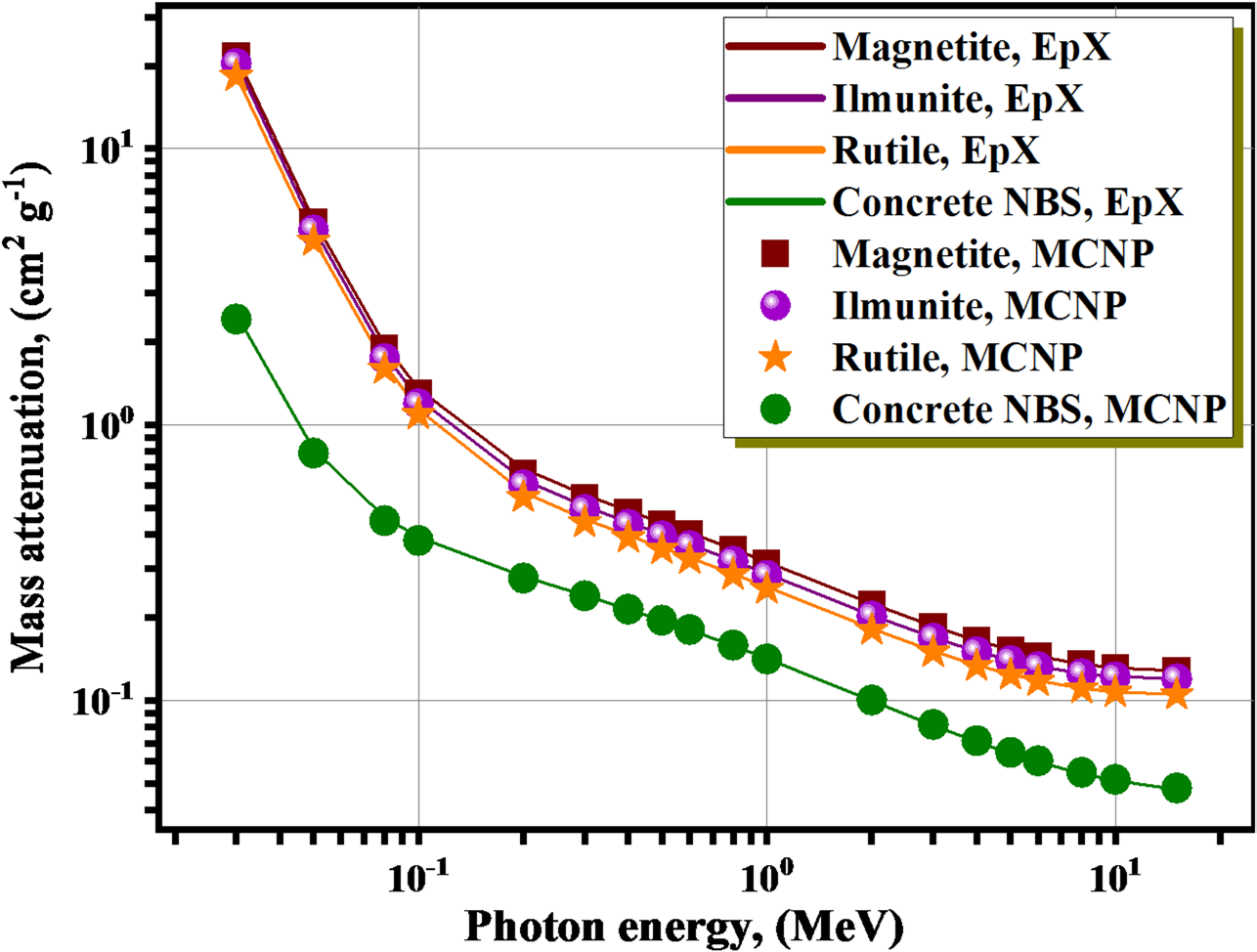
The variation of µ values vs. the γE for the studied materials.

The linear attenuation coefficient (µ, cm^−1^) values for the three natural minerals (Magnetite, Rutile, and Ilmenite) and concrete NBS within the γE of 0.03 ≤ γE ≤ 15 MeV have been evaluated using MCNP and EpX software to assess the radiation barrier features explored at Fig. 2. The relative differences (φ, %) among the mass attenuation coefficient (µ_m_, cm^2^g^−1^) outcomes of MCNP and EpX were calculated shown in Table 2. In the photon transport calculations, the maximum φ remained < 5.333%. The larger φ at low energy (e.g., at 0.08 MeV, φ for magnetite and NBS concrete are 3.511% and 2.839%, respectively) reflects the dominance of the photoelectric effect (σpe ​∼ Z^n^/E^3^), which accentuates differences in Z_ef_ and composition between Fe-rich magnetite and silicate-based concrete. As the energy increases, Compton scattering—governed primarily by electron density—becomes the leading interaction, and pair production contributes above 1.022 MeV; both mechanisms reduce material-specific contrasts per unit mass, so φ decreases (e.g., at 15 MeV, φ is only 0.266% for magnetite and 1.090% for NBS concrete). To verify that this trend is physical rather than code-dependent, we compared our MCNP and EpiXs predictions with NIST-XCOM mass attenuation coefficients for the same compositions/densities. The XCOM-based relative differences show the same monotonic decrease with energy and agree with our results within the combined uncertainties^45,46^.

**Table 2:** The evaluated values of mass Attenuation (µ_m_) using MCNP and EpX software for the investigated materials.

Energy (MeV)	Mass attenuation coefficient (cm^2^ g^−1^)											
	Magnetite			Ilmunite			Rutile			Concrete NBS		
	MCNP	EpX	φ %	MCNP	EpX	φ %	MCNP	EpX	φ %	MCNP	EpX	φ %
0.03	4.2072	4.2301	0.543	4.3134	4.3576	1.015	4.3222	4.3320	0.225	1.0728	1.1149	3.773
0.05	1.0531	1.0731	1.861	1.0749	1.0956	1.887	1.0910	1.1105	1.757	0.3508	0.3706	5.333
0.08	0.3649	0.3781	3.511	0.3679	0.3819	3.680	0.3754	0.3888	3.444	0.1994	0.2052	2.839
0.1	0.2509	0.2616	4.078	0.2519	0.2627	4.110	0.2564	0.2666	3.816	0.1690	0.1738	2.725
0.2	0.1293	0.1345	3.820	0.1282	0.1336	4.030	0.1284	0.1337	3.919	0.1234	0.1252	1.489
0.3	0.1054	0.1082	2.584	0.1044	0.1072	2.683	0.1042	0.1069	2.527	0.1064	0.1068	0.401
0.4	0.0930	0.0946	1.790	0.0920	0.0937	1.820	0.0917	0.0933	1.703	0.0952	0.0951	0.058
0.5	0.0845	0.0856	1.286	0.0836	0.0847	1.327	0.0833	0.0843	1.175	0.0868	0.0867	0.224
0.6	0.0779	0.0787	1.062	0.0771	0.0779	1.047	0.0768	0.0775	0.957	0.0803	0.0800	0.304
0.8	0.0684	0.0688	0.541	0.0677	0.0681	0.536	0.0673	0.0677	0.619	0.0706	0.0702	0.562
1	0.0609	0.0617	1.357	0.0602	0.0611	1.504	0.0597	0.0607	1.608	0.0628	0.0631	0.435
2	0.0433	0.0436	0.670	0.0428	0.0431	0.724	0.0425	0.0429	0.807	0.0446	0.0443	0.619
3	0.0358	0.0361	0.619	0.0356	0.0358	0.630	0.0353	0.0355	0.628	0.0364	0.0361	0.831
4	0.0319	0.0320	0.475	0.0318	0.0319	0.462	0.0314	0.0316	0.510	0.0318	0.0315	0.895
5	0.0295	0.0296	0.377	0.0295	0.0296	0.443	0.0291	0.0292	0.406	0.0288	0.0286	0.816
6	0.0279	0.0281	0.436	0.0281	0.0282	0.392	0.0277	0.0277	0.314	0.0269	0.0266	1.018
8	0.0262	0.0263	0.329	0.0265	0.0266	0.305	0.0260	0.0261	0.161	0.0244	0.0241	1.054
10	0.0254	0.0255	0.305	0.0258	0.0258	0.245	0.0252	0.0253	0.142	0.0230	0.0227	1.094
15	0.0248	0.0249	0.266	0.0254	0.0254	0.205	0.0247	0.0248	0.154	0.0214	0.0212	1.090

**Fig. 3:**
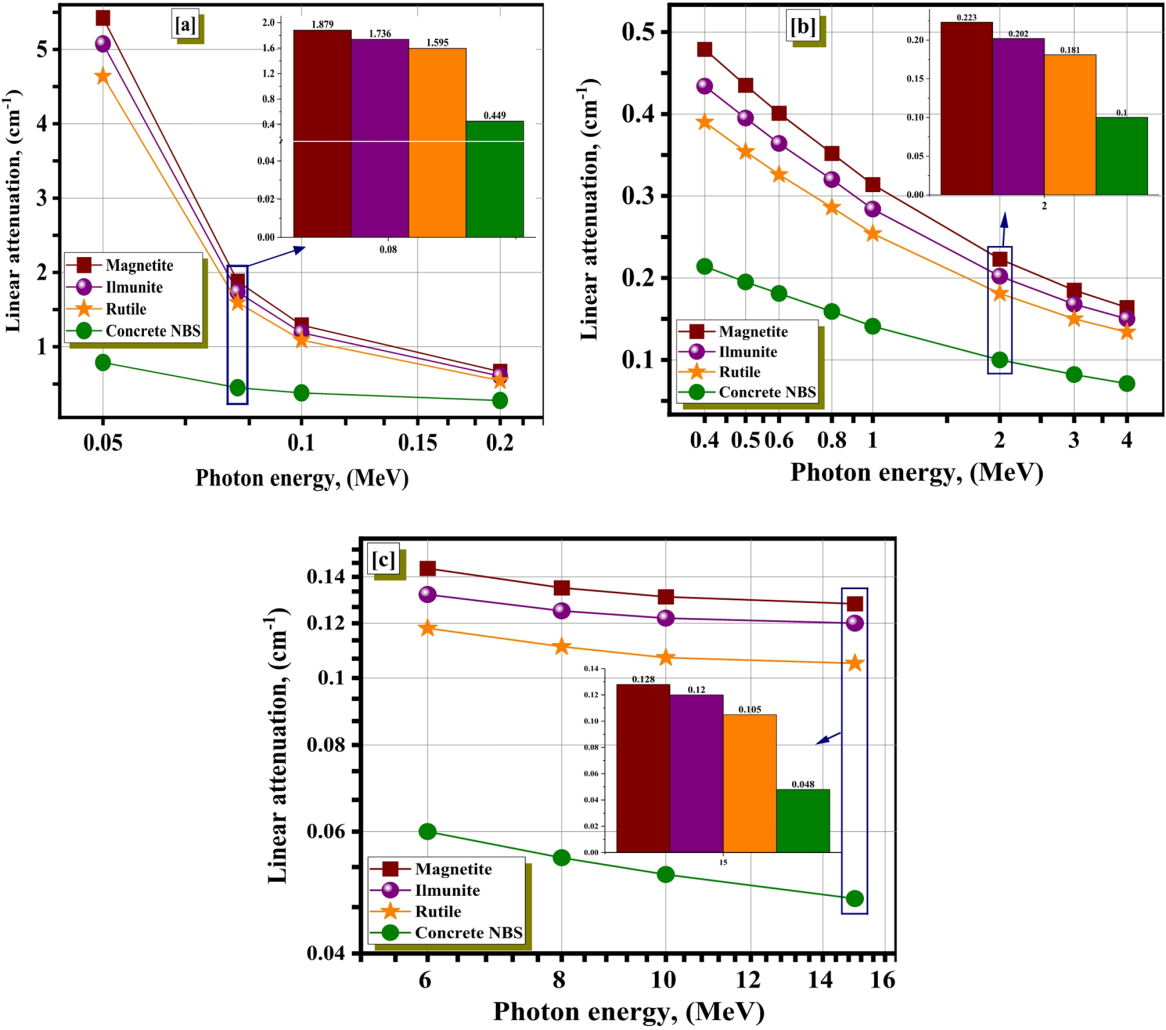
The µ vs. the γ energy at (**a**) PHE region, (**b**) COS region, and (**c**) PAP region for the investigated materials.

Figure 3a shows that the photoelectric effect (PHE), whose cross-section (δ) varies with γE^3.5–4.5^, causes a significant decrease in the linear attenuation (µ) values for all chosen shield materials^47,48^. Thus, the enhancement of γE values, which is linked to a corresponding decrease in γ-e^−^ interactions and µ values, resulted in a large reduction of the interaction δ. The improvement of the practical γE at 0.03 ≤ γE ≤ 0.2 MeV causes a solid effective lessening movement from 21.667 to 0.666 cm^−1^ for magnetite, from 18.370 to 0.546 cm^−1^ for rutile, from 20.359 to 0.605 cm^−1^ for ilmenite and from 2.414 to 0.278 cm^−1^ for concrete NBS. When compared to the other materials in this region, magnetite often possesses the highest µ values out of the others. In addition, the enrichment of γE greater than 200 keV results in an exponential drop in the µ within the photons energy interval of 300 keV ≤ γE ≤ 4 MeV, as demonstrated in Fig. 3b. The Compton scattring (COS) connected to δ changes with γE^−1^ is responsible for the exponential decline that has been observed on the graph^49,50^. Because of their larger velocity, atoms in the material with a higher γE are less likely to interact with one another. This can be linked to the fact that γE is higher. As a result, the possibility of absorption reduces in conjunction with the enhanced probability of γs scattering as the value of γE increases. A smooth decrease in the δ was related with the higher γE amounts, which be followed by a slight reduction in the µ values. This has been conjoined with decreasing the quantity of photon-electron interactions. In accordance with increasing the γE values at 200 keV ≤ γE ≤ 4 MeV, the µ values decreased for magnetite, rutile, ilmenite and concrete NBS from 0.543 to 0.164 cm^−1^, from 0.443 to 0.134 cm^−1^, from 0.493 to 0.150 cm^−1^, and from 0.239 to 0.071 cm^−1^, respectively. In addition, there is a little decrease because of the pair production processes (PAP) interaction with δ variations with $\:{{\upgamma\:}\text{E}}^{2}$^51,52^. The µ from 0.152 to 0.128 cm^−1^ for magnetite, from 0.124 to 0.105 cm^−1^ for rutile, from 0.139 to 0.120 cm^−1^ for ilmenite, and from 0.065 to 0.048 cm^−1^ for concrete NBS at 5 ≤ γE ≤ 15 MeV as realized in Fig. 3c. In comparison to the other materials, the magnetite mineral exhibits the greatest µ values, showing the greatest effective natural material in terms of blocking radiation at low, medium, and high energies within the spectrum due to its a high density (5.15 g.cm^−3^) and high concentration of FeO_3_ content (84.04% by weight).

**Fig. 4:**
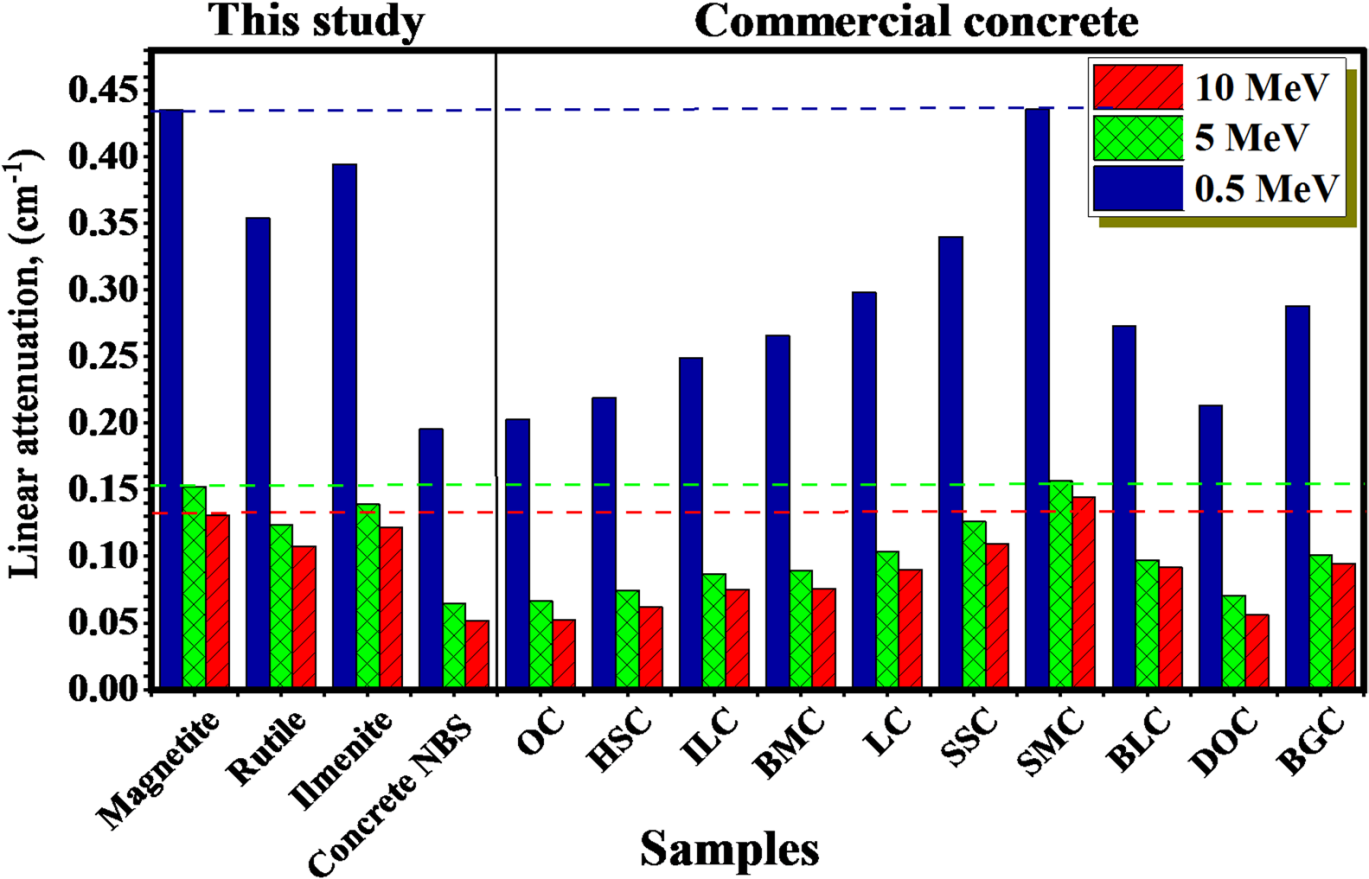
The µ, cm^−1^ vs. γ energy for the natural minerals and concrete NBS compared with reference concert compounds.

Figure 4 represents a comparison of the µ values of the studied compounds to those of commercial concretes^53,54^ at selected γE = 0.5, 5 and 10 MeV‎, the µ values of the Magnetite mineral compound exhibit greater values than those of compared concrete materials except the SMC material. It indicates that the studied Magnetite natural mineral has a good potential against radiation shielding.

**Fig. 5:**
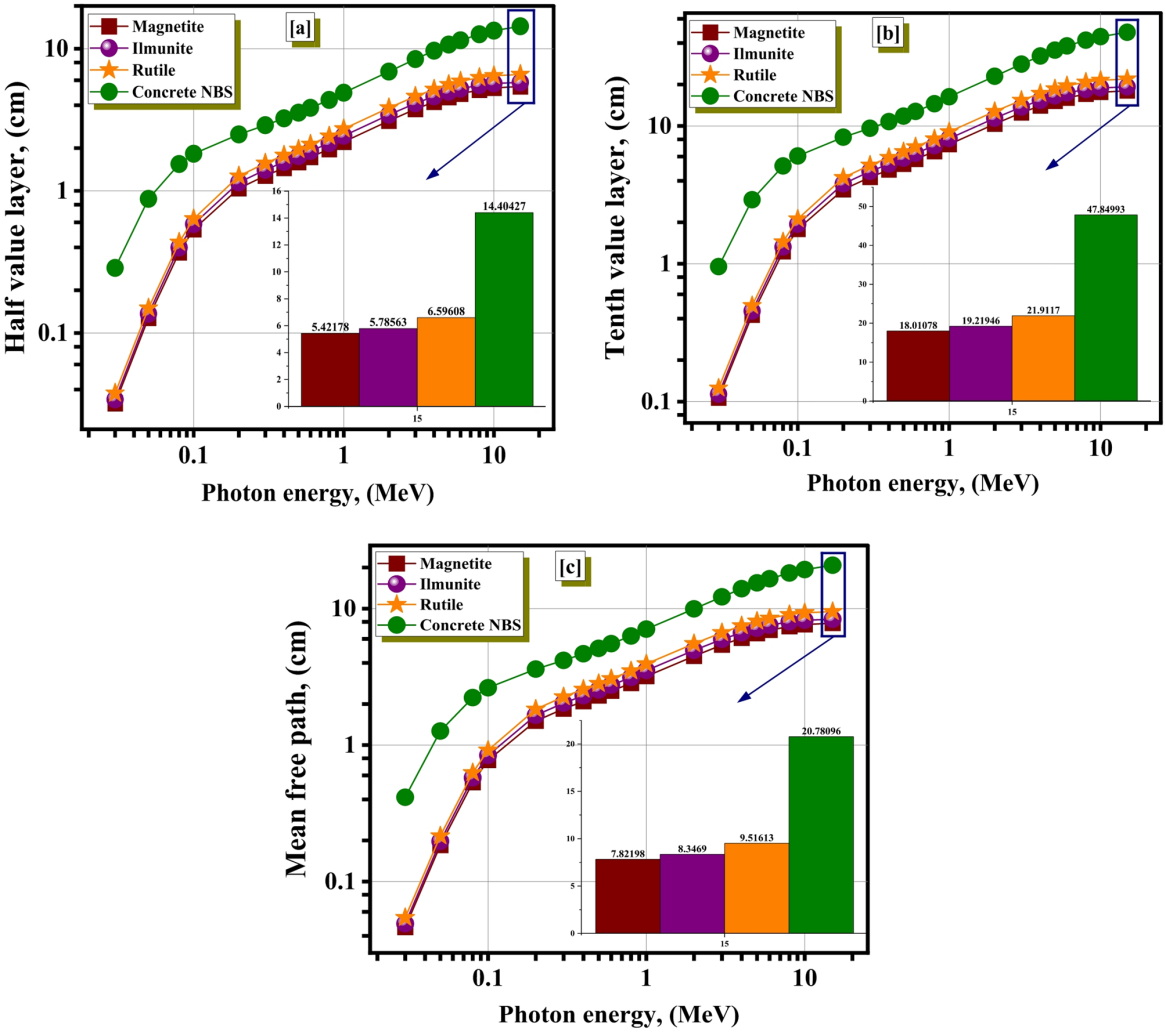
The variation of the H_0.5_ (**a**), the T_0.1_ (**b**), and the MFP (**c**) for the studied compounds with the γE.

As illustrated in Fig. 5a, the H_0.5_ values for magnetite, rutile, ilmenite, and concrete NBS ranged from 0.031 to 5.421 cm, 0.037 to 6.596 cm, 0.0340 to 5.785 cm, and 0.287 to 14.404 cm, respectively, predicated from the simulated µ values for a γ-ray at 30 keV ≤ γE ≤ 15 MeV. Additionally, as illustrated in Fig. 5b, the T_0.1_ values ranged from 0.953 to 47.849 cm, 0.106 to 18.0107 cm, 0.125 to 21.911 cm, and 0.113 to 19.219 cm for concrete NBS, magnetite, rutile, and ilmenite, respectively, with the γE values rising among 0.03–15 MeV. Along the same lines as the variation in µ values that was discussed before, the MFP values exhibit a rising pattern. These values ranged from 0.046 to 7.821 cm for magnetite, from 0.054 to 9.516 cm for rutile, from 0.049 to 8.346 cm for ilmenite, and from 0.414 to 20.780 cm for ilmenite, as can be observed in Fig. 5c. Because it had the lowest MFP values, magnetite was a material that was better suitable for applications involving radiation attenuation.

**Fig. 6:**
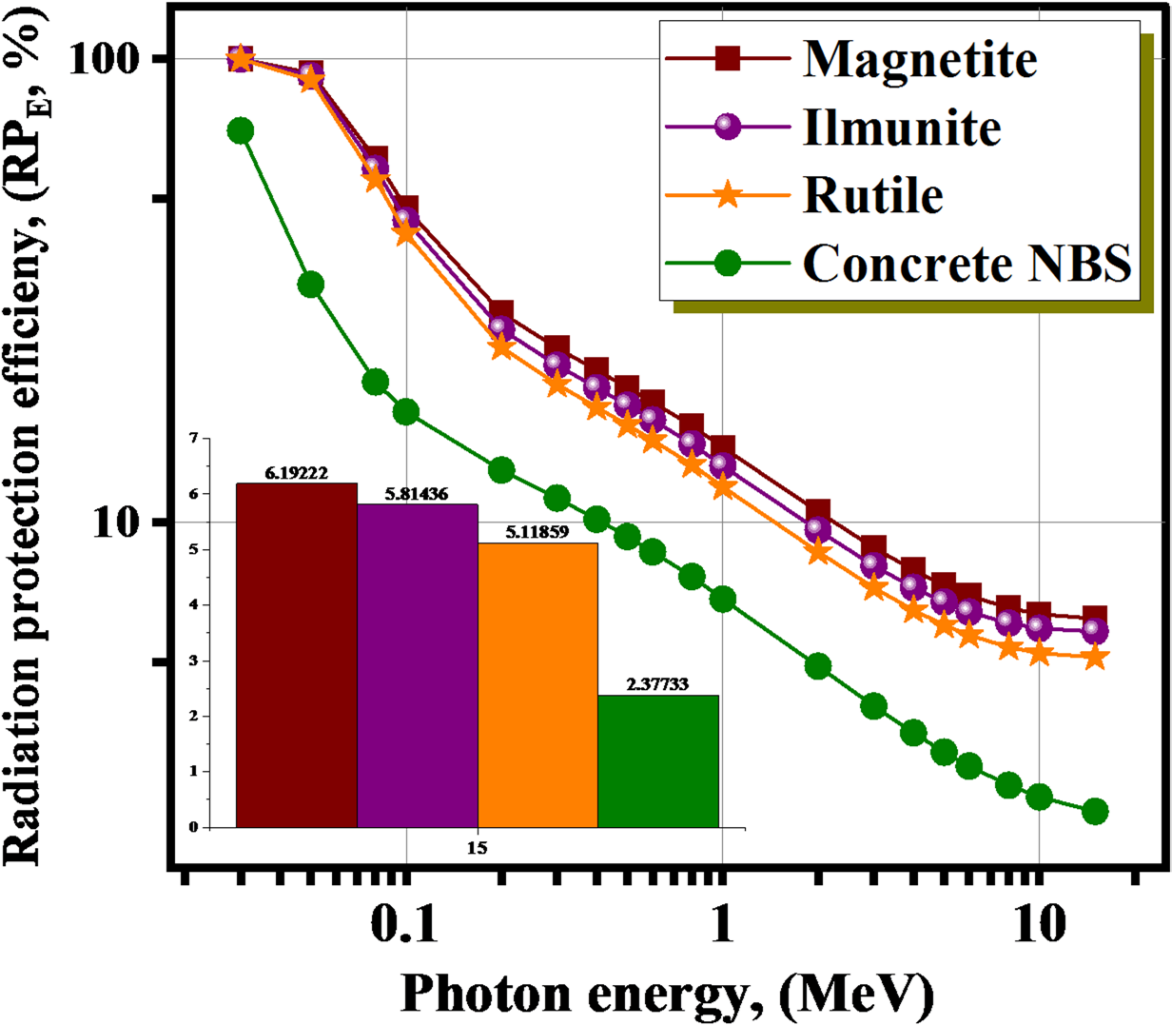
The RP_E_ for the investigated minerals with the γE.

The RP_E_ for the investigated minerals was affected by the γE and the elements doping, as illustrated in Fig. 6. At extremely low energies (γE ≤ 0.03 MeV), the RP_E_ values are extremely close to 100%. When the γE was increased, the penetrating strength of the photons that were supplied also increased, which resulted in a large fall in the RP_E_ levels. Consequently, when the energy of the γ-photon is increased, the γ-electron interactions that occur within the materials of minerals that are being examined are lowered. The relative particle energy (RP_E_) of the minerals materials that were examined is negatively impacted at lower interaction between photons and electrons. This is because increasing of the scattered photons number, which decreases the RPE. For example, The RP_E_ values reduced from ≈ 99.998, 99.996, 99.990%, and 70.088% at 0.030 MeV for all the investigated minerals to 6.192%, 5.814%, 5.119%, and 2.377%, at 15 MeV for the Magnetite, Rutile, Ilmenite, and concrete NBS materials, respectively at 0.030 ≤ γE ≤ 15 MeV. The consequences approve a great protection capability for the Magnetite mineral.

**Fig. 7:**
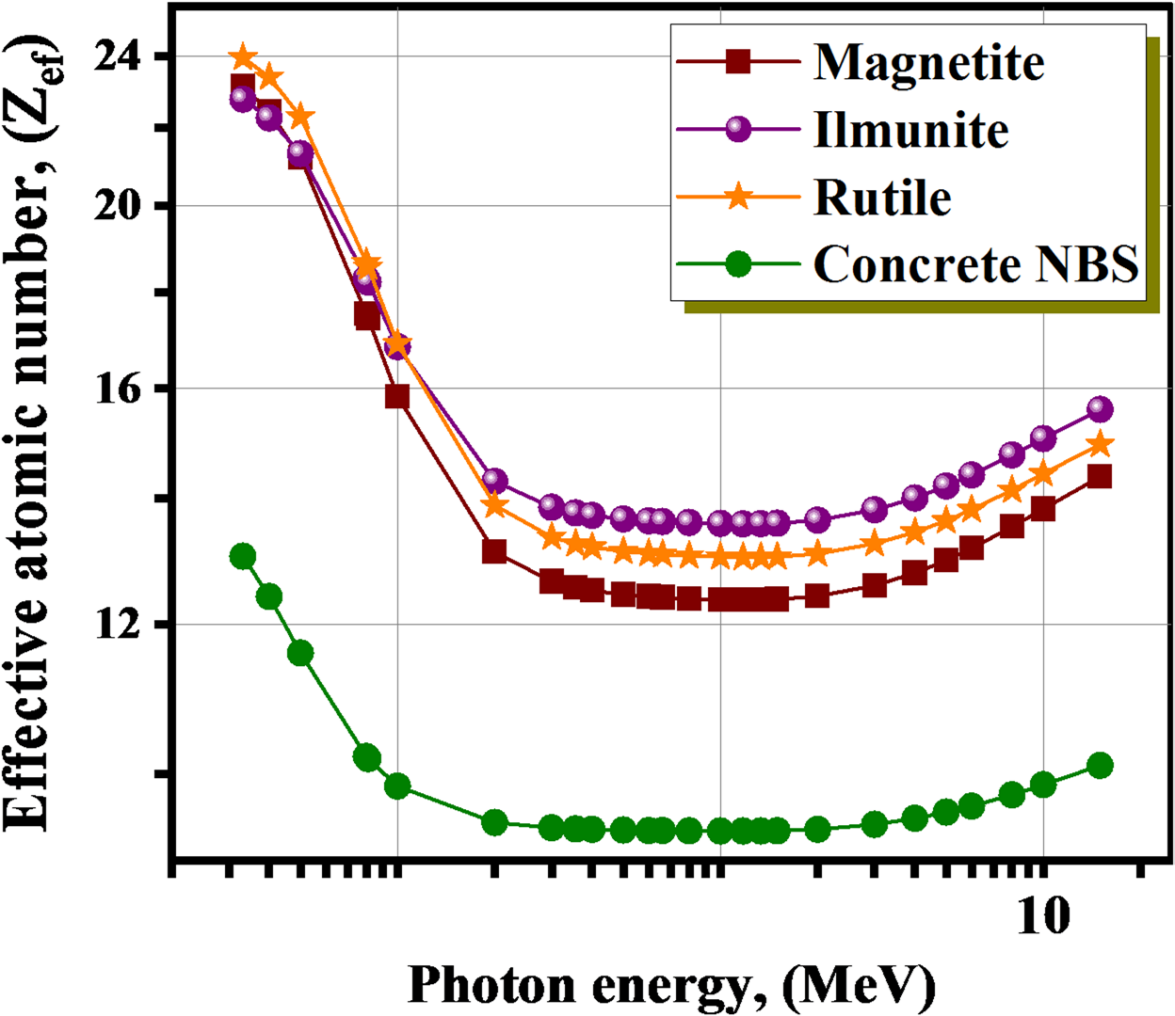
Variation of the Z_ef_ for the investigated minerals with the γE.

When it comes to a composite, the effective atomic number, which is sometimes referred to as Z_ef_ is an important metric with real-world applications in a variety of domains, including engineering, physics, and current technology. An explanation for the variety of characteristics exhibited by a material can be found in the value of Z_ef_. Figure 7 shows an example of the connection between the Z_ef_ and the γE for the materials under investigation. Usually, an increase in the Z_ef_ value indicates a greater quantity of radiation interaction within a given material, especially through the Compton effect and the photoelectric effect^55,56^. Consequently, high Z_ef_ value materials may better block high-energy γ-radiation^57,58^. At interesting energy spectrum, the range of Z_ef_ varied from 23.400 to 14.370, 24.160–14.950, 22.970–15.600, and 13.330–10.100 for Magnetite, Rutile, Ilmenite, and concrete NBS, respectively. The concrete NBS has the minimum Z_ef_ values, while the magnetite has the maximum Z_ef_ values among 15 keV ≤ γE ≤ 15 MeV due to higher content with a FeO_3_ (84.040 wt.%).

**Fig. 8:**
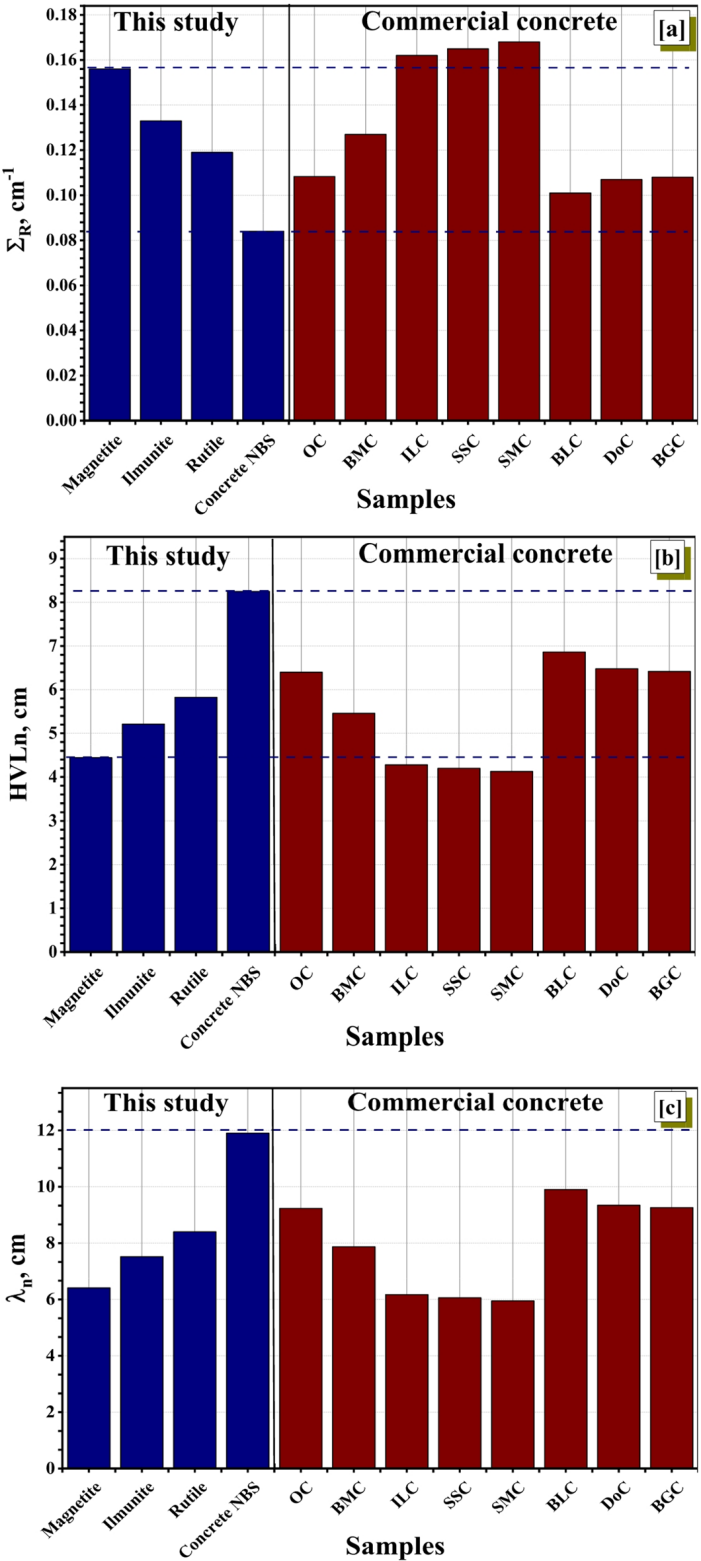
(**a**) The Σ_R_, (**b**) the HVL_n_, and (**c**) the λ_n_ of the investigated minerals and some commercial concrete materials.

As can be observed in Fig. 8a, the data that has been presented reveals that the fast neutrons removal cross-section (∑_R_) for the investigated minerals that Magnetite, Rutile, Ilmenite, and concrete NBS, were 0.156, 0.133, 0.119, and 0.084 cm^−1^, respectively. The ∑_R_ of Magnetite was highest than that of the others due to the higher concentration of the light element (oxygen). As well, the ∑_R_ of the investigated minerals have been compared with commercial concrete shield materials (e.g., ordinary (OC), hematite-serpentine (HSC), ilmenite limonite ‎ (ILC), basalt-magnetite (BMC), Ilmenite (IC), Steel-scrap (SSC), Steel magnetite (SMC), traditional mix (DoC), sand/limonite (BLC), and boron carbide/sand/goethite mix (BGC))^40,59,60^ as illustrated at Fig. 8a. With the exception of ILC, SSC, and SMC concretes, the magnetite mineral’s ∑_R_ value was found to be higher than that of the commercial concrete materials under comparison. Figure 8b,c displays the HVL_n_ and λ_n_ for the chosen natural minerals materials and other commercial concretes. Furthermore, the magnetite mineral had the lowest HVLn and λn values according to the simulated ∑_R_ values. It is reasonable to presume that the magnetite being studied has superior neutron shielding properties.

**Fig. 9:**
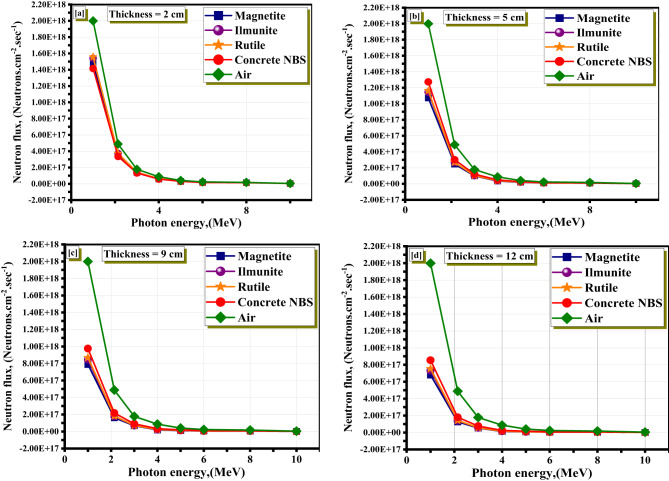
Neutrons spectra at different thicknesses for the studied minerals.

In addition, the neutrons spectra at different thicknesses (2 cm, 5 cm, 9 cm, and 12 cm) for the studied minerals beside air proves in Fig. 9. The same trend of the high neutron attenuation performance of the Magnetite mineral due to either the elemental composition or the sample thickness. The increase in the sample thickness increases the neutron’s attenuation performance. The Ilmenite, Rutile, and concrete NBS came after that, respectively. From the plotted neutron spectra, it is evident that Magnetite shows the most significant reduction in neutron intensity across all energy ranges, particularly at higher thicknesses. At 12 cm, the neutron flux is almost completely suppressed, indicating efficient absorption and scattering capabilities. This behavior is attributed to the high density of Magnetite and its elevated concentration of elements like iron and oxygen, which are known for their effective neutron-moderating and absorbing properties. In contrast, concrete NBS exhibited the least attenuation, especially in the fast neutron energy range (above 1 MeV), suggesting its limited effectiveness in high-energy neutron environments. The neutron attenuation profiles confirm that Magnetite is a highly suitable candidate for shielding applications where fast neutron flux reduction is critical. This includes nuclear reactor containment walls, spent fuel storage areas, and medical radiotherapy vaults that utilize neutron-producing devices.

**Fig. 10:**
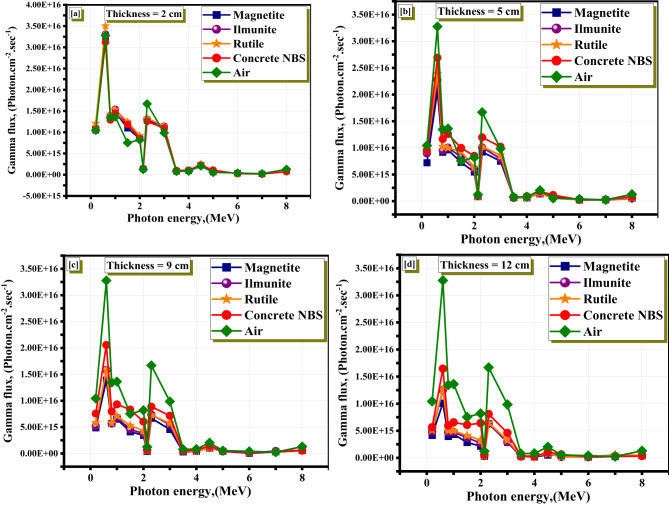
The primary and secondary gamma spectra at different thicknesses for the studied minerals due to neutrons attenuation.

Looking at the γ-ray spectra, which include both the main γ-rays from the source and any secondary γ-rays from nuclear reactions with the shield components, taken after the shield materials were conditioned with different thicknesses, it is clear that the peak at about 2.2 MeV is mainly due to the H(n,γ)D reaction^16,61^, declines significantly as the thickness of the magnetite mineral increases. The former reaction’s cross-section grows significantly larger as the neutron energy decreases. When neutrons are thermalized, it becomes a big deal^61,62^. Having a composite shield that absorbs fast neutral neutrons and then emits extremely energetic hard γ-rays at the same time is not acceptable in environments that are subject to mixed radiation. This is a very important consideration to take into account when working with shielding applications, especially in environments that are characterised by mixed radiation. However, when compared to other materials, the H(n,γ)D reaction is significantly suppressed when the thickness of the magnetite increases^61^. When compared to the other mixtures that were investigated, the magnetite mineral had the lowest flux at 2.2 MeV photon energy. This was observed at the maximum thickness of 12 cm, as shown in Fig. 10. By examining the data in the attached gamma spectrum table, it is evident that Magnetite exhibits the lowest photon flux at 2.2 MeV for all thicknesses (2 cm to 12 cm), compared to Ilmenite, Rutile, and NBS. At 12 cm thickness, the reduction in flux becomes most significant, confirming Magnetite’s superior attenuation performance in mixed radiation fields. This behavior is highly favorable for shielding design, especially in environments where both neutron and photon radiation are present simultaneously, such as in nuclear reactors or radioactive waste management facilities. A material that not only attenuates primary photons and neutrons but also limits the production of secondary high-energy γ-rays is crucial to ensure overall radiation safety.

## Conclusion

This research discusses the common minerals, magnetite, rutile, ilmenite, and concrete NBS to focus on the radiation shielding performance against energetic gamma, neutrons, and the secondary gamma emitted from neutrons interactions. The investigation used the Monte-Carlo code and EpiXs software in the γE between 0.03 and 15 MeV. The conclusions were considered as follows:

- The µ for the studied minerals was from 21.667 to 0.128 cm^−1^ for magnetite, 18.370 to 0.105 cm^−1^ for rutile, 20.359 to 0.120 cm^−1^ for ilmenite and 2.414 to 0.048 cm^−1^ for concrete NBS.
- The linear attenuation coefficient (µ) order is: Magnetite > ilmenite > rutile > concrete NBS.
- The Magnetite minerals has the maximum linear attenuation values and the minimum H_0.5_, T_0.1_, and MFP values.
- The fast neutron removal cross-section (∑_R_) were 0.156, 0.133, 0.119, and 0.084 cm^−1^ for Magnetite, Rutile, Ilmenite, and concrete NBS, respectively.
- The Magnetite mineral had the highest fast neutron removal cross-section value (∑_R_) and lowest HVL_n_ and λ_n_ values. It was made possible by its high density and high concentration of light components (oxygen).
- The Magnetite mineral could be effective in nuclear installations with secondary gamma rays.
- The magnetite mineral showed the lowest main/secondary gamma-rays and neutrons impact in case of increasing the sample thickness, which indicates promising performance for use as radiation shielding in complex radiation fields.

## Data Availability

All data generated or analyzed during this study are included in this published article.
